# 3D reconstruction of coronary artery bifurcations from coronary angiography and optical coherence tomography: feasibility, validation, and reproducibility

**DOI:** 10.1038/s41598-020-74264-w

**Published:** 2020-10-22

**Authors:** Wei Wu, Saurabhi Samant, Gijs de Zwart, Shijia Zhao, Behram Khan, Mansoor Ahmad, Marco Bologna, Yusuke Watanabe, Yoshinobu Murasato, Francesco Burzotta, Emmanouil S. Brilakis, George Dangas, Yves Louvard, Goran Stankovic, Ghassan S. Kassab, Francesco Migliavacca, Claudio Chiastra, Yiannis S. Chatzizisis

**Affiliations:** 1 Cardiovasclar Biology and Biomechanics Laboratory, Cardiovascular Division, University of Nebraska Medical Center, Omaha, 68105 USA; 2 StudioGijs, Daendelsstraat 40, 5018 ES Tilburg, The Netherlands; 3 Biosignals, Bioimaging and Bioinformatics Laboratory (B3-Lab), Department of Electronics, Information and Bioengineering, Politecnico di Milano, 20133 Milan, Italy; 4 Department of Cardiology, Teikyo University Hospital, Tokyo, 173-0003 Japan; 5 Department of Cardiology, National Hospital Organization Kyushu Medical Center, Fukuoka, 810-0065 Japan; 6 Department of Cardiovascular Sciences, Fondazione Policlinico Universitario A. Gemelli IRCCS Università Cattolica del Sacro Cuore, 00168 Rome, Italy; 7 Minneapolis Heart Institute, Minneapolis, 55407 USA; 8 Department of Cardiovascular Medicine, Mount Sinai Hospital, New York City, 10029 USA; 9 Institut Cardiovasculaire Paris Sud, 91300 Massy, France; 10 Department of Cardiology, Clinical Center of Serbia, 11000 Belgrade, Serbia; 11 California Medical Innovation Institute, San Diego, CA 92121 USA; 12 Laboratory of Biological Structure Mechanics (LaBS), Department of Chemistry, Materials and Chemical Engineering “Giulio Natta, Politecnico di Milano, 20133 Milan, Italy; 13 PoliToBIOMed Lab, Department of Mechanical and Aerospace Engineering, Politecnico di Torino, 10129 Turin, Italy

**Keywords:** Interventional cardiology, Biomedical engineering, Computational models, Computational platforms and environments, Image processing, Programming language, Software

## Abstract

The three-dimensional (3D) representation of the bifurcation anatomy and disease burden is essential for better understanding of the anatomical complexity of bifurcation disease and planning of stenting strategies. We propose a novel methodology for 3D reconstruction of coronary artery bifurcations based on the integration of angiography, which provides the backbone of the bifurcation, with optical coherence tomography (OCT), which provides the vessel shape. Our methodology introduces several technical novelties to tackle the OCT frame misalignment, correct positioning of the OCT frames at the carina, lumen surface reconstruction, and merging of bifurcation lumens. The accuracy and reproducibility of the methodology were tested in n = 5 patient-specific silicone bifurcations compared to contrast-enhanced micro-computed tomography (µCT), which was used as reference. The feasibility and time-efficiency of the method were explored in n = 7 diseased patient bifurcations of varying anatomical complexity. The OCT-based reconstructed bifurcation models were found to have remarkably high agreement compared to the µCT reference models, yielding r^2^ values between 0.91 and 0.98 for the normalized lumen areas, and mean differences of 0.005 for lumen shape and 0.004 degrees for bifurcation angles. Likewise, the reproducibility of our methodology was remarkably high. Our methodology successfully reconstructed all the patient bifurcations yielding favorable processing times (average lumen reconstruction time < 60 min). Overall, our method is an easily applicable, time-efficient, and user-friendly tool that allows accurate and reproducible 3D reconstruction of coronary bifurcations. Our technique can be used in the clinical setting to provide information about the bifurcation anatomy and plaque burden, thereby enabling planning, education, and decision making on bifurcation stenting.

## Introduction

Coronary artery bifurcations represent unique anatomical locations in the epicardial coronary tree with increased susceptibility to coronary artery disease^1,2^. Specific anatomic features of bifurcations, including the angle and diameter of the main vessel (MV) and side branch (SB), have significant impact on the local hemodynamic milieu and subsequent propensity to atherosclerosis^3,4^. The bifurcation anatomy and extent of disease are substantial determinants of bifurcation stenting strategies and clinical outcomes^5^. Three-dimensional (3D) representation of the bifurcation anatomy and disease burden could help us better appreciate the anatomical complexity of bifurcation disease and optimize our stenting strategies.

Dedicated single-modality 3D reconstruction of coronary bifurcations can be performed with either 3D quantitative coronary angiography (3D QCA) or coronary computed tomography angiography (CTA)^6–8^. However, both these modalities have major limitations: 3D QCA cannot provide the correct geometrical information of the bifurcation lumen due to the inherent assumptions related to the use of two 2D angiographic planes. Nevertheless, 3D QCA provides accurate details on the 3D course of the bifurcation centerline^9,10^. Coronary CTA is limited by heart and lung motion artifacts and coronary calcifications, resulting in the exclusion of a descent portion of patients^11^. Hybrid multi-modality 3D reconstruction of bifurcations based on the fusion of intravascular ultrasound (IVUS) or optical coherence tomography (OCT) of the MV only with coronary CTA or invasive angiography has been described^6,12,13^. These approaches have limitations mostly related to the accuracy of SB reconstruction. Notably, the use of different imaging modalities for MV and SB reconstruction results in inaccuracies in the reconstruction of the geometrically sensitive and clinically important bifurcation carina and SB. Also, using invasive imaging (IVUS) for the reconstruction of MV and non-invasive imaging (CTA) for the reconstruction of SB is not easily applicable in the clinical setting.

In this work, we build upon the current state-of-the-art and propose a novel strategy for the 3D reconstruction of coronary bifurcations based on the fusion of invasive coronary angiography—which provides the bifurcation centerline—with OCT of both MV and SB. The goals of our study are (1) to describe the methodology for 3D reconstruction of coronary bifurcations, and (2) to systematically test the accuracy, feasibility, and reproducibility of the method in patient-specific silicone bifurcation models, as well as in patient coronary artery bifurcations with varying degrees of disease.

## Methods

All methods were carried out in accordance with the relevant guidelines and regulations. The angiograms and OCT data were obtained from a clinical trial, named PROPOT (Randomized Trial of the Proximal Optimization Technique in Coronary Bifurcation Lesions). The study was approved by the ethics committee of Teikyo University (IRB approval number 15-159-2) and informed consent was obtained from all subjects.

### Silicone models

**Figure 1:**
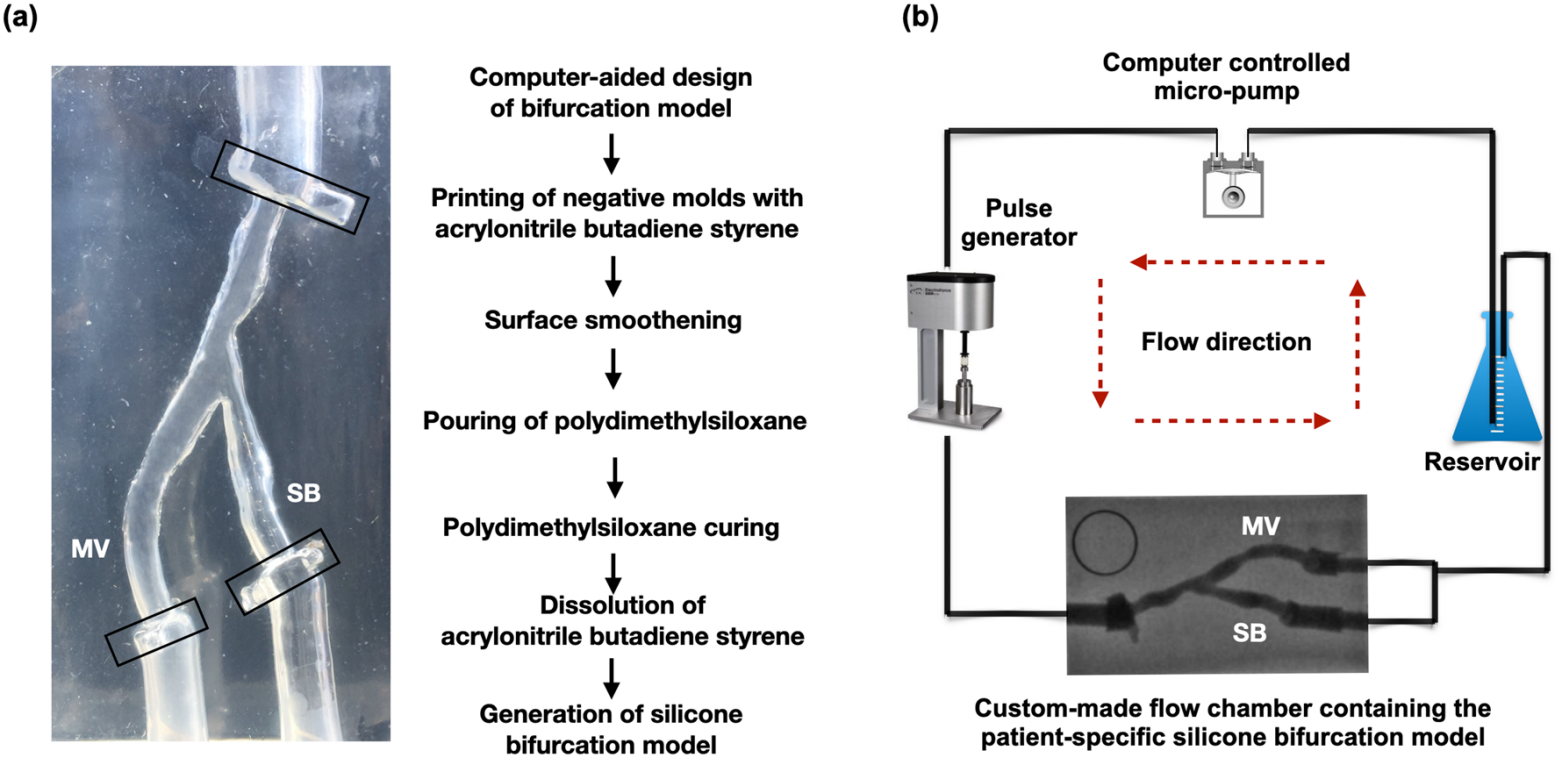
Patient-specific silicone bifurcation models and bioreactor flow circuit. (**a**) Generation of the silicone bifurcation model and a representative example with the fixed markers (black boxes) at the distal and proximal end, (**b**) Bioreactor flow circuit showing the angiographic image of the bifurcation model in the flow chamber.

Five patient-specific silicone models of coronary artery bifurcations (Supplementary Information Table S1) were created, using our in-house developed technique. The bifurcation geometries were 3D reconstructed from human coronary angiograms during the diastolic phase of the cardiac cycle, using commercially available software (3D CAAS Workstation 8.2, Pie medical imaging, Maastricht, The Netherlands; Fig. 1a). To demarcate the region of interest and stabilize the silicone models during the imaging procedures, tube-like extensions and fixed markers were added at the inlet and outlet of the reconstructed bifurcations using a computer-aided design software (Rhinoceros 6, Robert McNeel & Associates, Seattle, USA). For every model, a negative mold was designed and converted to stereolithography (STL) file. The STL file was 3D printed with acrylonitrile butadiene styrene material using the Stratasys Dimension Elite 3D printer (Stratasys, Rehovot, Israel) at a resolution of 178 μm. Acetone vapor was used to produce a smooth inner surface. The molds were stored in room temperature for 8–12 hours and cleaned with distilled water and dried. Polydimethylsiloxane was mixed with its curing agent and then placed into a vacuum for a total of 1 h and 30 min to remove the air bubbles. Subsequently, polydimethylsiloxane was poured into the dry clean molds, which were placed in the vacuum to remove any remaining air bubbles and then put in the oven for polydimethylsiloxane curing for 48 h at the temperature of 65 °C. After curing, the silicone models were put in an acetone beaker, which was placed in an ultrasonic cleaner (Branson 1800, Cleanosonic, Richmond, USA) for 8–10 h to dissolve all acrylonitrile butadiene styrene material.

### Contrast-enhanced micro-computed tomography (μCT) imaging

All the bifurcation models were imaged with μCT (Skyscan 1172 version 1.5, Antwerp, Belgium) using the following parameters: image pixel size 26.94 μm, voltage 100 kV, current 100 μA, and slice thickness 27 µm. To visualize the lumen borders effectively, iodinated contrast media (37%) was injected into the lumen. The bifurcations were 3D reconstructed from the μCT images using a 3D medical imaging software (Mimics 22.0, Materialise, Leuven, Belgium) and smoothened using Meshmixer (Autodesk Research, New York, USA).

### Bioreactor flow circuit for invasive imaging procedures

The silicone-based bifurcation models were placed in a custom-made flow chamber. Polyvinyl chloride tubing was connected at the inlet and outlet ports of the silicone models. A bioreactor circuit was connected to the inlet and outlet of the flow chamber, allowing circulation of 1000 ml of deionized water at a steady flow-rate of 100 ml/min at room temperature (Fig. 1b). All the bifurcation models were imaged with angiography and OCT imaging of both MV and SB.

### 3D QCA for 3D reconstruction of bifurcation centerline

**Figure 2:**
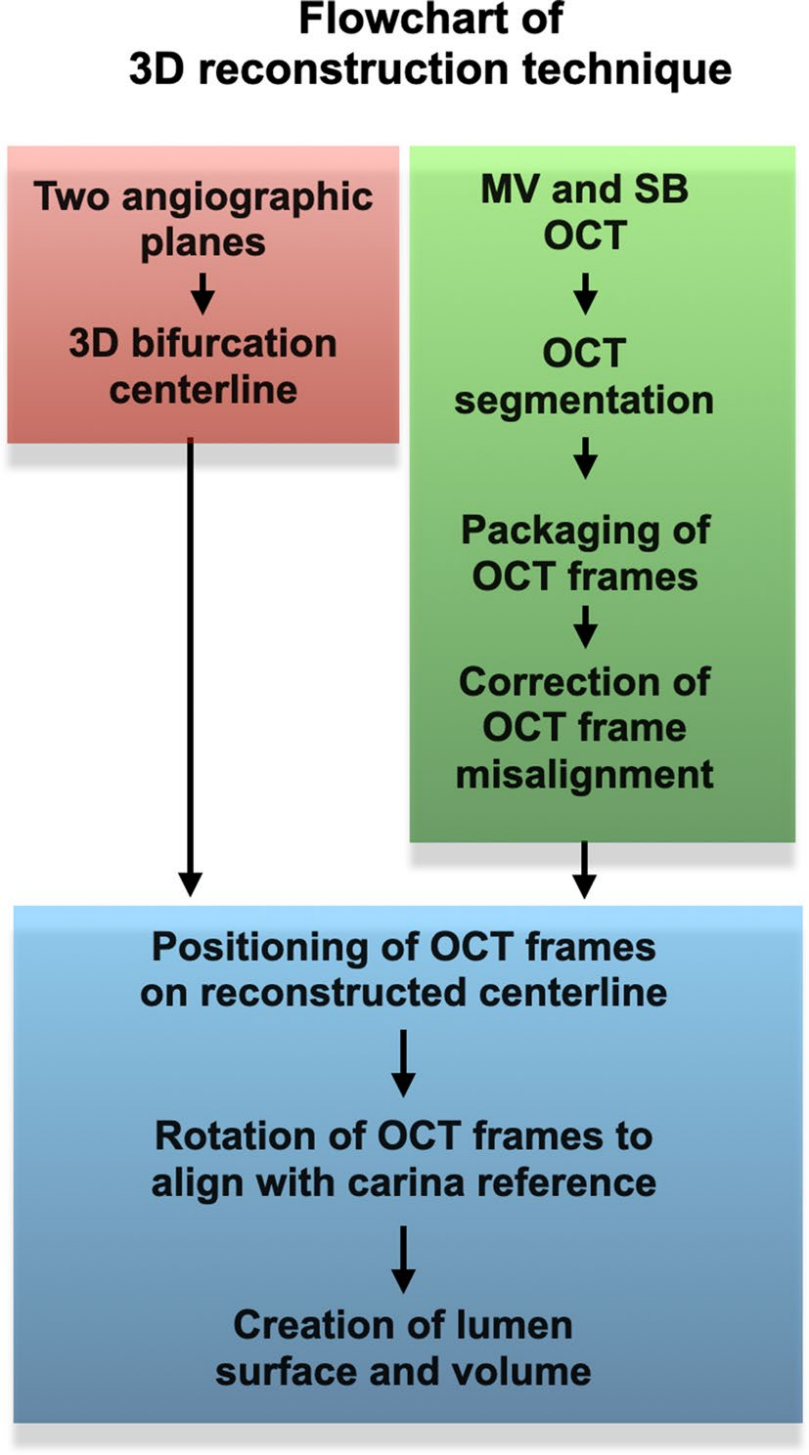
Flowchart of 3D reconstruction of coronary artery bifurcation.

**Figure 3:**
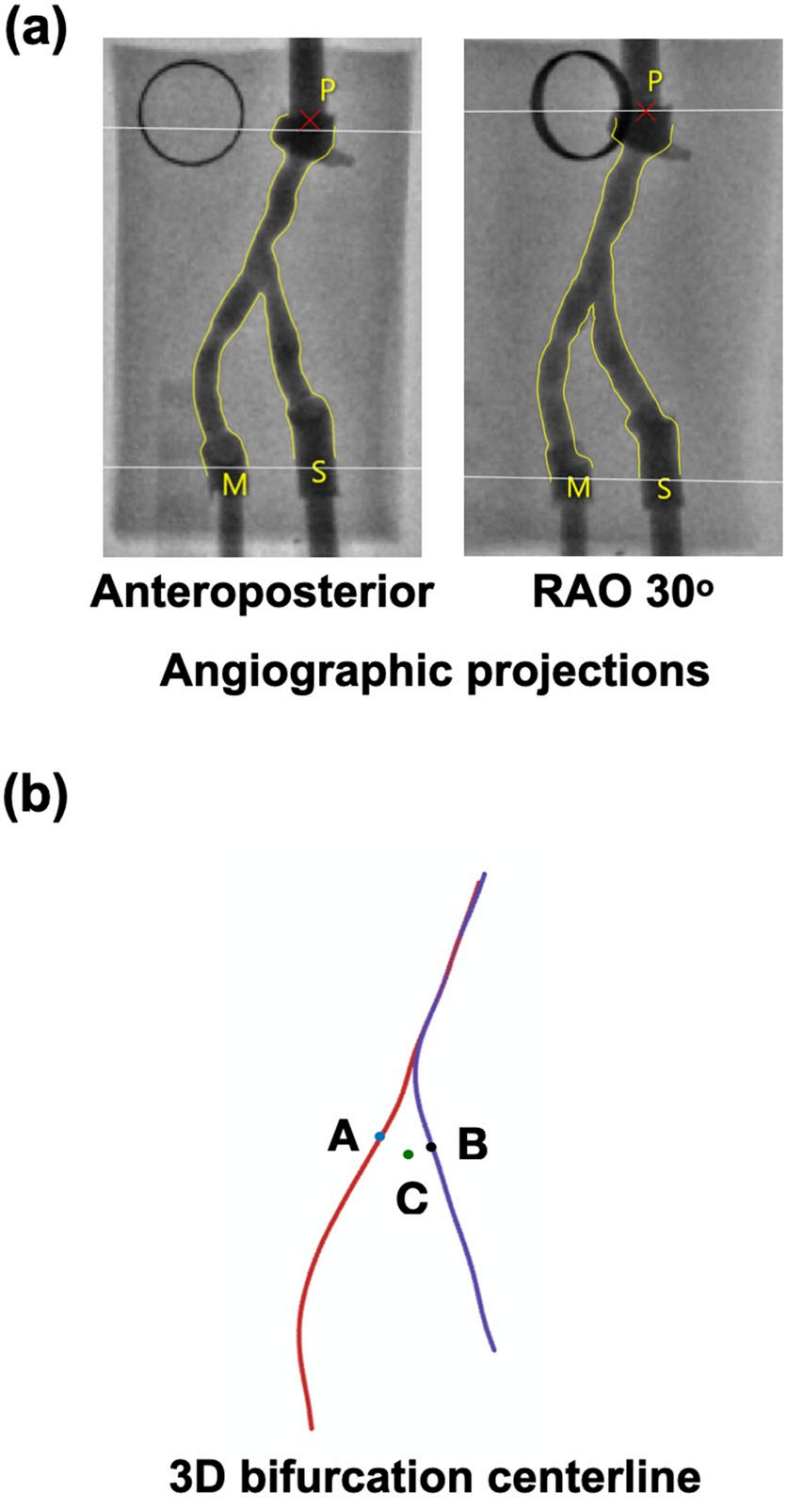
Angiograhic image processing. (**a**) Two angiographic projections. (**b**) 3D reconstruction of the bifurcation centerline. Note that points A and B correspond to the carina points on the MV and SB centerlines, respectively, whereas point C (green) corresponds to the carina reference (carina location).

**Figure 4:**
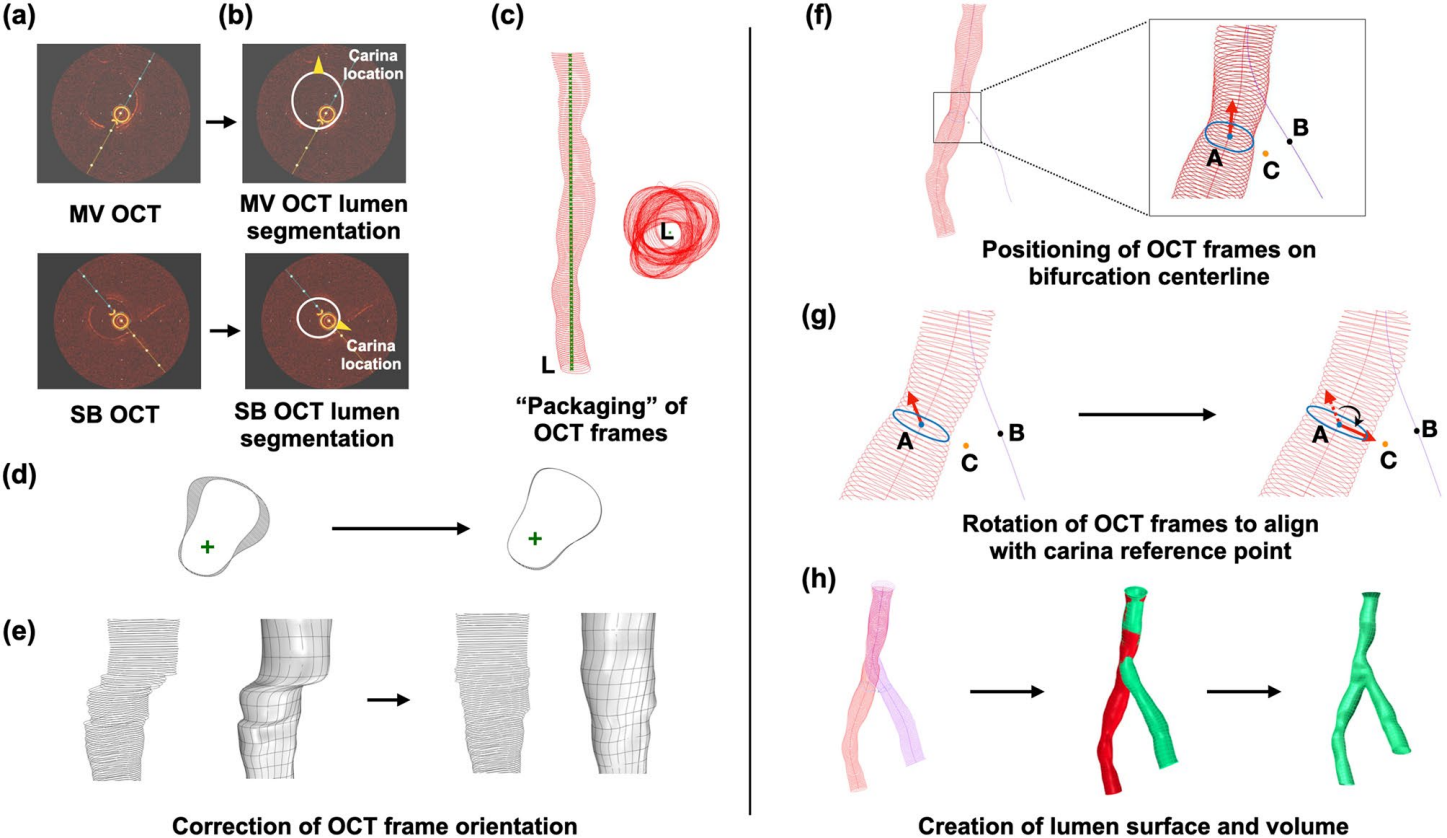
3D reconstruction of bifurcation lumen from OCT. (**a** and **b**) Main vessel (MV) and side branch (SB) OCT frames at the carina. The carina location in each frame is indicated by a yellow arrow, (**c**) OCT frames “packaging” along the straight catheter centerline (L) shown in longitudinal and axial view, (**d**) Correction algorithm for OCT frame orientation errors. Two successive unmatched OCT frames are displayed. The catheter center (i.e., frame rotation center) is denoted by the green cross. The overlapping outside areas are hatched. The concept of the correction algorithm was to rotate two successive OCT frames around the catheter center (green cross) until they are aligned, and the outside frame overlap is minimal. When the outside overlap area exceeded a certain threshold, the script rotated the mismatched frames in 0.5° increments to minimize the overlapping area. (**e**) Illustration of the effect of the correction algorithm in a real patient case. After orientation correction, the significant gaps were eliminated, resulting in a continuous and smooth reconstructed model, (**f**) Positioning of the OCT frames on the bifurcation centerline with reference to carina points A and B on the MV and SB centerlines, respectively (SB frames are not shown to avoid overlapping). In the carina frame (blue), the direction from the catheter center to the carina location was set as reference direction (red arrow), (**g**) The carina OCT frame (blue) was positioned on the respective site along its centerline and rotated until its direction reference (red arrow) was aligned with the carina reference (orange point C). Then, all the rest of the OCT frames were simultaneously rotated by the same angle like the carina frame, (**h**) Reconstruction of the final 3D bifurcation model using T-spline. In the proximal MV, the shape of the reconstructed MV and SB were similar, but not exactly the same. Since OCT catheter pullback in MV is straighter than in SB, the proximal MV OCT frames were chosen to reconstruct the overlapping proximal MV segment.

The flowchart for the 3D reconstruction of the bifurcation model is shown in Fig. 2, and the detailed steps in Figs. 3 and 4. Angiography of the bifurcation models was performed at two projections with at least 30° difference in viewing angles (Fig. 3a). In each projection, the lumen of the segment of interest was manually detected, and the bifurcation carina was set as a common reference location (carina reference). The 3D replica of the bifurcation models was created in CAAS and exported to VMTK (Orobix, Bergamo, Italy) for the extraction of MV and SB centerlines. On each centerline, a carina point was found according to the carina reference projected to the centerline (Fig. 3b).

### Acquisition and segmentation of OCT

OCT imaging of the MV and SB was obtained using the OPTIS Integrated System (Abbott, Chicago, IL, USA; Fig. 4a). The OCT catheter (Dragonfly, Optis Imaging Catheter) was advanced through a 6F guiding catheter and pulled back (automatic triggering by saline without contrast) at a speed of 36 mm/s (5 frames/mm) for 75 mm, covering the entire length of MV and SB from the distal to the proximal fixed marker (Fig. 1a). Lumen segmentation of the OCT frames was carried out semi-automatically using echoPlaque 4.0 (INDEC Medical Systems, Los Altos, CA, USA; Fig. 4b).

### OCT processing for bifurcation lumen reconstruction

The detailed steps of the bifurcation lumen reconstruction are illustrated in Fig. 4. Briefly, the segmented OCT frames were imported into Grasshopper 3D (visual programming language and environment that runs within the Rhinoceros 3D) and packaged in a straight line along the catheter center (Fig. 4c). The OCT frame misalignment was corrected with an in-house script (Fig. 4d and e). The correctly aligned OCT frames were positioned perpendicularly on the respective bifurcation centerline passing through the centroid of each frame (Fig. 4f). The OCT frame at the carina (blue frame in Fig. 4f) was positioned at the carina point (blue point A in Fig. 4f), and the rest of the frames were positioned in a specific location along the centerline according to the known distance between them. The frames were then rotated to align with the carina reference (orange point C in Fig. 4g). The primary surfaces of MV and SB were created and served as a reference for the creation of a final uniform, smooth, and continuous bifurcation surface using the T-spline method (Fig. 4h).

### Validation

The 3D OCT reconstructed bifurcation models were compared with the corresponding 3D μCT reconstructed ones, using the latter ones as reference. First, the 3D OCT and μCT reconstructed models were co-registered using the carina and fixed markers (Fig. 1a). The following metrics were used for the method comparison studies: (1) Lumen area, (2) Lumen shape, and (3) Bifurcation angles. To minimize possible biases, different operators performed the 3D reconstruction from OCT, 3D reconstruction from μCT, and comparison between OCT- and μCT-based models.

#### Lumen area

Serial cross-sections were identified every 2 mm along the lumen of the MV and SB in the OCT and μCT models. We noted a consistent difference in lumen area between OCT vs. μCT, attributed to the fact that OCT pullback was performed in a saline environment, resulting in underestimation of the true lumen dimensions. To quantitatively assess the differences between OCT and μCT imaging, we used a silicone-based tube with known lumen dimensions. The tube was imaged with OCT (pulled back under the same conditions with the bifurcation models) and μCT. The median lumen area of the OCT-reconstructed tube was 6.62 mm^2^, interquartile range (IQR) 6.42 to 6.89 mm^2^, and of the μCT-reconstructed tube was 7.96 mm^2^, IQR 7.94 to 8.05 mm^2^; Supplementary Information Fig. S1]. To account for the systemic and consistent discrepancies of lumen size between OCT and μCT, the lumen areas were normalized using the z-score^14^.

#### Lumen shape

In each cross-section, we calculated the ratio of the maximum distance between the two furthest points of the circumference (distance X), and the maximum length that was perpendicular to distance X (distance Y). Assuming an oval-shaped lumen, the ratio of distance Y/distance X was used as a marker of lumen shape.

#### Bifurcation angles

The following three bifurcation angles were calculated using an in-house algorithm: Angle A between the proximal MV and SB, angle B between the distal MV and SB, and angle C between the distal and proximal MV (see detailed description in Supplementary Information Fig. S2).

### Reproducibility

To calculate the reproducibility of the OCT-based 3D reconstruction method, the same operator re-reconstructed all the silicone models. To minimize the recall bias, the reconstructions were performed three months apart. The reconstructed models at the two-time points were compared in terms of lumen area, lumen shape, and bifurcation angles.

### Feasibility and processing times in human bifurcations

**Figure 5:**
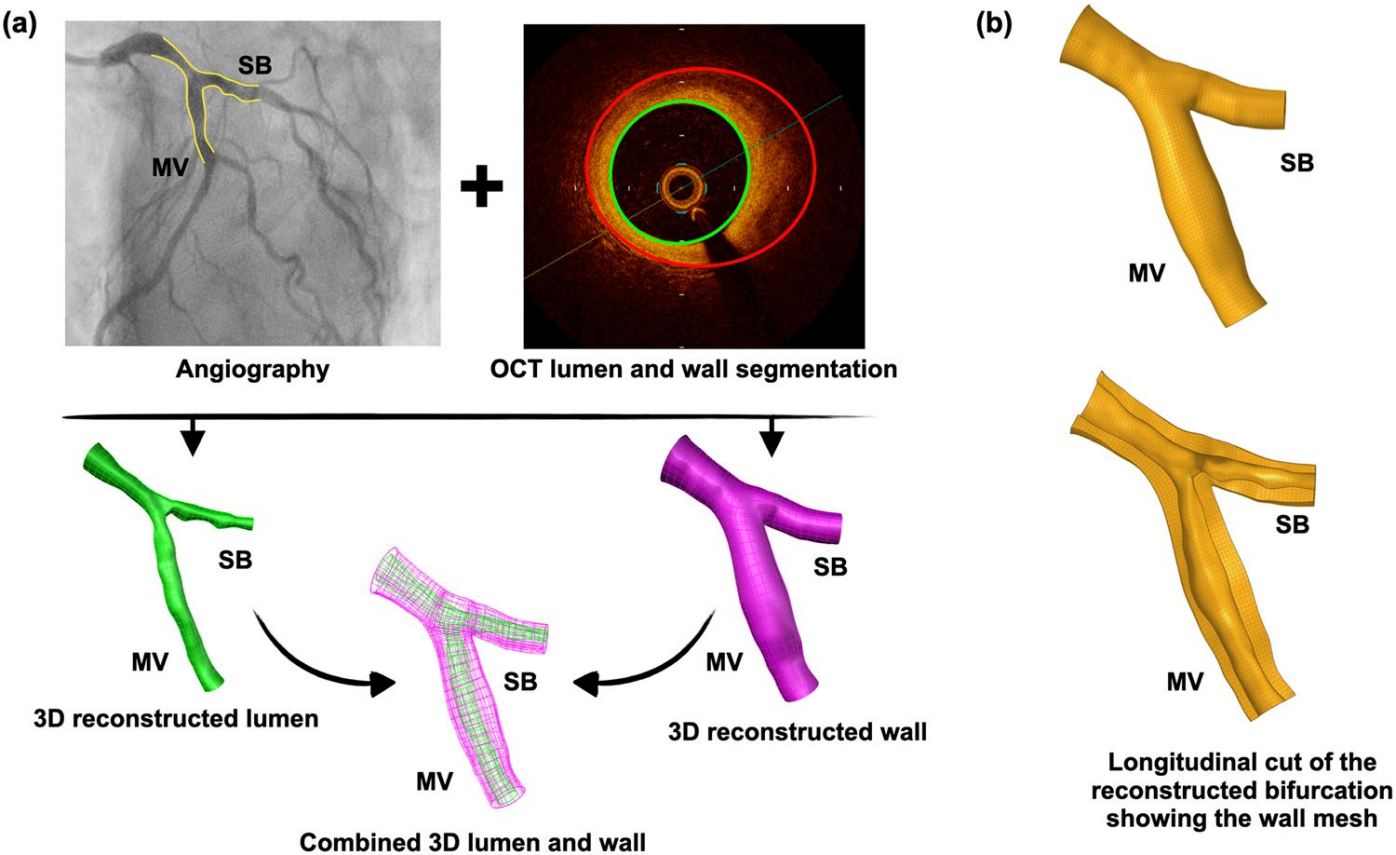
A representative example of a 3D reconstructed patient bifurcation lumen and wall. (**a**) The fusion of angiography with OCT resulted in the 3D reconstructed bifurcation model, including lumen and wall, (**b**) Meshed bifurcation ready for finite element analysis.

The feasibility and processing time of our method were assessed in n = 7 patient coronary artery bifurcations with varying degrees of disease and calcification (Supplementary Information Table S1). OCT and angiography data were acquired according to the protocols mentioned above. Both lumen and wall were 3D reconstructed following the steps of our proposed methodology. We followed a step-wise approach for the delineation of the outer borders in OCT images. Our approach worked successfully in > 95% of images and involved the following steps: (1) In case of ill-defined outer wall borders, we were limiting the outer wall at the margin of the complete signal loss (Supplementary Information Fig. S3a), (2) In case the margin of complete signal loss could not be identified in < 180 degrees of vessel circumference, we were interpolating the visible outer wall border (Supplementary Information Fig. S3b), and (3) In case the margin of complete signal loss could not be identified in > 180 degrees of vessel circumference, we were discarding that particular OCT frame and segmenting an adjacent frame following the same steps 1–2. After segmenting the vessel wall in OCT images, the wall was 3D reconstructed following the same steps as with the lumen (applying the same frame orientation correction and frame rotation as with the lumen), and the bifurcation was built by combining the lumen and wall interface (Fig. 5a). The reconstructed bifurcations could be meshed with hexahedral elements (Fig. 5b). To assess the overall time-efficiency of our method, we calculated the processing time for each step in each of the seven cases.

### Statistical analyses

Statistical analyses were performed with the statistical package GraphPad Prism 8.0 (GraphPad Inc., San Diego, CA, USA). Continuous variables were expressed as median (IQR). The lumen areas of OCT and μCT models were normalized by calculating the z-score as (absolute area-µ)/σ with µ representing the mean area and σ the standard deviation of the mean. The method comparison and reproducibility studies were performed with linear regression and Bland–Altman analysis. P-value < 0.05 was considered as the level of significance.

## Results

### Validation

#### Lumen area

**Figure 6:**
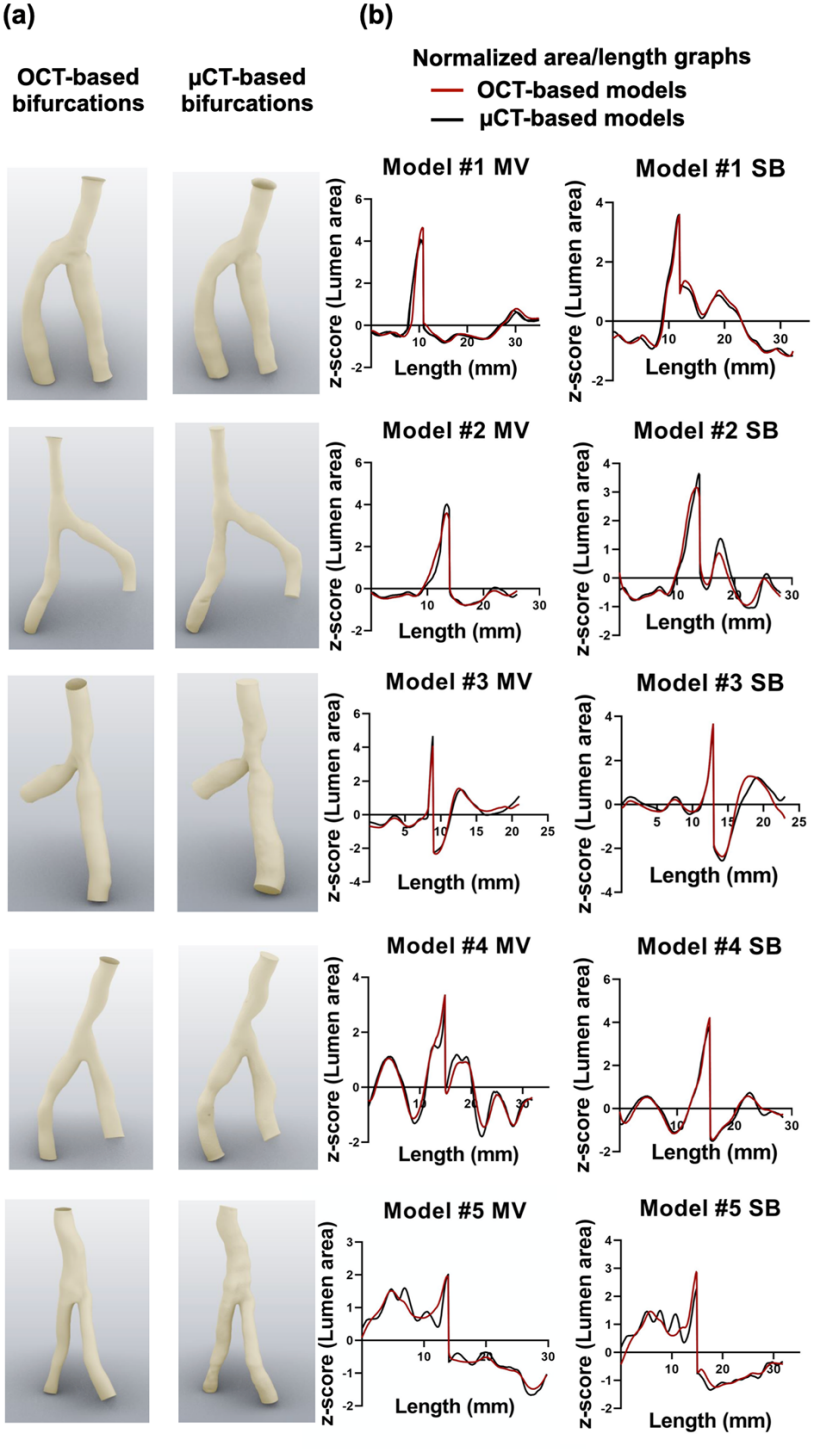
Comparison between OCT-based and μCT-based 3D reconstruction of silicone bifurcation models. (**a**) OCT- and μCT-reconstructed models, (**b**) Normalized lumen area/length graphs. The length is from lumen proximal to distal.

All n = 5 silicone models were successfully 3D reconstructed (Fig. 6a). The normalized (z-score) lumen areas of the 3D reconstructed bifurcations from 3D OCT vs. μCT showed high agreement in the normalized area/length graphs (Fig. 6b). Linear regression analysis showed r^2^ values between 0.91 and 0.98, slopes close to one, and intercepts close to zero (Table 1).

**Table 1:** Comparison between OCT- and μCT-reconstructed silicone models: Linear regression analysis of the normalized lumen areas (z-score) and median with interquartile range for lumen shape; MV: main vessel, SB: side branch**.**

Bifurcation	Branch	Lumen area		Lumen shape			
		r^2^	Linear regression equation	OCT median	OCT 25th, 75th percentile	µCT median	µCT 25th, 75th percentile
#1	MV	0.92	y = 0.96x − 00	0.87	0.73, 0.92	0.85	0.73, 0.93
	SB	0.98	y = 0.99x − 00	0.90	0.73, 0.94	0.87	0.72, 0.95
#2	MV	0.96	y = 0.98x − 00	0.82	0.74, 0.88	0.83	0.76, 0.93
	SB	0.95	y = 0.97x − 00	0.87	0.77, 0.94	0.91	0.79, 0.94
#3	MV	0.96	y = 0.98x − 00	0.93	0.89, 0.97	0.93	0.86, 0.97
	SB	0.91	y = 0.95x + 00	0.91	0.79, 0.93	0.93	0.82, 0.94
#4	MV	0.93	y = 0.96x − 00	0.86	0.75, 0.95	0.87	0.70, 0.93
	SB	0.96	y = 0.98x − 00	0.86	0.80, 0.93	0.90	0.75, 0.94
#5	MV	0.96	y = 0.98x + 00	0.77	0.59, 0.91	0.73	0.57, 0.92
	SB	0.92	y = 0.96x − 00	0.90	0.67, 0.93	0.89	0.57, 0.95

#### Lumen shape

The median ratio of maximum distances perpendicular to each other (distance Y/distance X) was 0.87 (0.85–0.90) for OCT and 0.88 (0.84–0.91) for µCT reconstructed bifurcation models (Table 1). Bland Altman analysis of the median ratios (distance Y/distance X) between 3D OCT and µCT models revealed a minimal mean difference of 0.002 (− 0.05 to 0.05), suggesting a high level of agreement (Fig. S4a).

#### Bifurcation angles

All three bifurcation angles (A, B, and C) showed a high level of agreement between the 3D OCT reconstructed bifurcation and the μCT reconstructed models (Table 2), suggesting the ability of our method to reconstruct the bifurcation carina accurately. The linear regression analysis for the three angles of all bifurcations showed r^2^ = 0.99, y = 1.03x-4.64 with *p* < 0.05 (Supplementary Information Table S2). The Bland–Altman analysis revealed an average angle difference of 0.004° (− 8.17° to 8.15°) (Supplementary Information Fig. S4a).

**Table 2:** Comparison of bifurcation angles between OCT- and μCT-reconstructed models.

Bifurcation	Angles (in degrees)					
	Angle A		Angle B		Angle C	
	3D OCT	µCT	3D OCT	µCT	3D OCT	µCT
#1	148.60	147.36	59.73	64.12	151.67	148.50
#2	141.24	138.77	69.90	73.22	148.85	148.01
#3	160.13	162.55	39.88	35.11	159.93	162.34
#4	152.76	154.95	54.41	50.33	152.82	154.73
#5	153.06	160.25	50.95	41.94	156.00	157.69

### Reproducibility

The comparison metrics for the reproducibility of our method are shown in Table 3 and Supplementary Information Tables S3 and S4. The lumen areas of the OCT reconstructed bifurcation models at two-time points showed very high agreement (r^2^ = 0.98; y = 0.96x + 0.19, *p* < 0.001). Bland Altman analysis showed mean differences in bifurcation angle of 0.004 (− 6.67 to 6.68), and lumen shape of 0.01 (− 0.25 to 0.23), suggesting the high reproducibility of our method (Supplementary Information Fig. S4b).

**Table 3:** Reproducibility of the OCT-based 3D reconstruction method: Linear regression comparing the lumen areas of the silicone models reconstructed twice by the same operator 3 months apart; MV: main vessel, SB: side branch**.**

Bifurcation			Lumen area	
	Branch	r^2^	Linear regression equation	*p* value
#1	MV SB	0.99 0.98	y = 1.00x − 0.31 y = 0.93x + 0.10	< 0.001 < 0.001
#2	MV SB	0.99 0.99	y = 1.00x − 0.27 y = 1.02x − 0.02	< 0.001 < 0.001
#3	MV SB	0.99 0.99	y = 0.97x − 0.21 y = 1.03x + 0.17	< 0.001 < 0.001
#4	MV SB	0.99 0.99	y = 0.98x + 0.12 y = 0.95x + 0.30	< 0.001 < 0.001
#5	MV SB	0.99 0.99	y = 0.97x + 0.08 y = 0.95x + 0.16	< 0.001 < 0.001

### Feasibility

All n = 7 patient models were successfully reconstructed with our 3D reconstruction algorithm. Figure 5a shows a representative example of a meshed 3D reconstructed bifurcation, including lumen and wall (the rest n = 6 cases are shown in Supplementary Information Fig. S5). Figure 5b shows the meshing of the reconstructed bifurcations. The processing time for each step, from image processing to final 3D lumen and wall reconstruction, are summarized in Table 4. The average time for the reconstruction of a patient bifurcation lumen was less than 1 h.

**Table 4:** Processing times for the OCT-based 3D reconstruction of patient coronary artery bifurcations (lumen only; n = 7).

Steps	Minutes
Step 1. Image pre-processing	
1. Angiography processing	15 ± 10
2. OCT segmentation	45 ± 15
Total time for image pre-processing	60
Step 2. 3D reconstruction of bifurcation lumen	
1. Data importing and parameter setting	20 ± 5
2. OCT frame error correction	2 ± 1
3. Localization and rotation of OCT frames on the centerline	2 ± 1
4. 3D reconstruction of primary bifurcation model	2 ± 1
5. 3D reconstruction of final bifurcation model	30 ± 5
Total time for 3D reconstruction of bifurcation lumen	56
Total time for whole process	116

## Discussion

In this study, we presented in detail a novel methodology for 3D reconstruction of coronary bifurcations that extends the current state-of-the-art. Using sophisticated bench and clinical data, we showed that our technique is accurate, reproducible, and time-efficient. Our technique can be used in the clinical setting to provide information about the bifurcation anatomy and plaque burden, thereby enabling clinical planning and decision making in the cardiac catheterization laboratory.

The 3D reconstruction of coronary bifurcations—particularly with calcified disease—has always been a challenging issue given the anatomical complexity of this coronary region that cannot be fully captured by a single imaging modality. 3D QCA provides only an approximation of the bifurcation carina, whereas coronary CTA is not widely applicable given its non-invasive nature, cardiac and lung motion and calcium blooming artifacts^11^. In principle, a hybrid (multi-modality) approach could potentially provide a more accurate representation of the bifurcation anatomy and disease. However, the methodologies published to date (OCT/angiography^15^ or IVUS/coronary CTA^6^) have been using different modalities for the reconstruction of MV and SB, resulting in geometrical inaccuracies of the carina. The optimal approach for 3D reconstruction of coronary bifurcation should have the following two characteristics: (1) Use of the same imaging modality for the imaging and reconstruction of MV and SB, (2) Use of high-resolution invasive imaging to capture the bifurcations (particularly the heavily calcified ones) coming to the cardiac catheterization laboratory. A recent brief conference report presented a technique for the reconstruction of bifurcations by merging coronary angiography and OCT of both MV and SB, but the method was essentially not validated, as it was compared to 3D QCA, which by no means is a gold-standard^16^. To the best of our knowledge, our methodology is the first in-depth and extensively validated report on using invasive imaging (OCT) of both MV and SB to reconstruct coronary bifurcations. The specific innovation of our methodology is based on the following three elements: First, the method used angiography, not only to rebuild the bifurcation centerline, which served as the “backbone” of the reconstruction but also to extract valuable information about the carina and OCT frame location. The smooth integration of OCT with the bifurcation “backbone” resulted in the accurate reconstruction of the vessel shape. Second, the method applied a correction algorithm to identify the optimal OCT frame orientation. Adequate OCT quality is of paramount importance for reliable lumen reconstruction. OCT is susceptible to cardiac motion artifacts secondary to the lack of ECG-gating, which can result in suboptimal OCT frame orientation, compromising the accuracy of the reconstructed vessel (Fig. 4)^17,18^. Previous attempts to address the frame orientation error used a branch-based correction algorithm^19,20^. However, these algorithms applied an approximate correction guided by two branch references, which were unable to correct the error at the precise spot. Our methodology followed a more sophisticated approach based on two principles: (1) The OCT frames could only rotate around the catheter center, which was fixed on the OCT frames, and (2) Consecutive OCT frames were nearly the same. With this approach, our algorithm resulted in a more accurate vessel shape reconstruction compared to other methodologies. Third, we used T-spline in the final reconstruction step, which provided a continuous and uniform “organic” substrate to combine the MV and SB surfaces accurately, particularly at the bifurcation. In contrast to the commonly-used non-uniform rational basis spline, T-spline has fewer control points and tessellation operations, can be locally refined, and has been widely used in the free-form design and reverse engineering^21^. Our methodology applied for the first time T-spline in bifurcations, resulting in very accurate reconstruction of the carina, which has been the “Achilles’ heel” of the previous methodologies^6,16,19,20,22–25^.

We performed a thorough validation of our methodology following a robust benchtop experiment with patient-specific silicone-based bifurcations incorporated in a perfusion circuit and imaged with μCT, which was used as gold-standard. For the comparison studies, we used a wide spectrum of morphometric indices including lumen size and shape, and bifurcation shape. The OCT-based reconstructed bifurcation models were found to have remarkably high agreement compared to the µCT reference models, yielding r^2^ values between 0.91 and 0.98 for the normalized lumen areas, and mean differences of 0.005 and 0.004 degrees for lumen shape and bifurcation angles, respectively. Likewise, the reproducibility of our methodology was remarkably high (Table 3 and Supplementary Information Tables S3 and S4).

Another key feature of our methodology is the excellent feasibility and versatility in a variety of real patient data and diseased bifurcation anatomies with varying degrees of calcification. Notably, our method was quite effective in reconstructing the wall along with the lumen, providing the framework for computational studies and a better understanding of plaque burden and complexity. Our method was time-efficient and user-friendly in part due to the use of a visual programming language tool (Grasshopper 3D). Unlike the traditional text-based code, such as Matlab, Grasshopper 3D allowed modularization, seamless workflow—even for operators without programming background—and semi-automation of the reconstruction process, with only minimal manual intervention for model checking and parameter setting.

Our methodology has several clinically important applications. The 3D reconstructed bifurcation can inform the proceduralists about the precise bifurcation anatomy, as well as the extent and severity of coronary artery disease. A better understanding of the disease burden can result in better procedural planning and outcomes. Moreover, the 3D reconstructed bifurcation lumen itself can be used for computational and experimental (bench) fluid dynamics studies to explore the role of flow in native coronary artery disease development and progression, as well as in stent restenosis and thrombosis^19,24,26,27^. Our methodology provides the accurate geometrical input needed for realistic computational fluid dynamics studies. Our technique can also provide the basis for finite element analysis and patient-specific computational simulations of bifurcation stenting. Furthermore, computational stenting simulations using patient-specific bifurcation anatomy and plaque properties, as well as realistic stent geometry, can provide personalized planning of stenting techniques^2,28^. Patient-specific bifurcation anatomies are also particularly relevant to the industry for the testing and development of new generation stents. Finally, the basic principles of our methodology can be translated to other invasive imaging modalities, e.g., IVUS or even non-invasive imaging, e.g., coronary CTA. As long as there is imaging data available to extract the lumen centerline and lumen/vessel wall borders, our methodology has the potential to perform well^6^.

This study has several limitations. First, we applied the z-score normalization for the comparison of lumen areas between OCT and µCT. This normalization was done to correct for the systemic dimension discrepancy between these modalities secondary to OCT imaging without contrast. However, this discrepancy did not affect the reconstruction method itself and was consistent between the two imaging modalities, suggesting negligible interference with the validation process. Second, the 3D reconstruction of the bifurcation wall was dependent on the outer wall segmentation in OCT images. Given OCT’s limited tissue penetration, the imaging of the outer wall borders can be suboptimal, limiting the applicability of OCT in wall reconstruction^29^. However, in our study, the outer wall was very meticulously delineated by an imaging expert (YSC), resulting in a faithful representation of the true arterial wall. Third, several commercially available software (i.e., CAAS, echoPlaque, VMTK) were used for the angiography and OCT image processing, which consumed about half of the total processing time. Further codes are under development to enable the visual programming language tool to perform the centerline extraction automatically and OCT segmentation, reducing the processing time dramatically and making our algorithm applicable in near real-time^30^.

In conclusion, in this work, we presented and extensively validated for the first time a methodology for accurate 3D representation of coronary artery bifurcations of varying anatomical complexity based on the fusion of angiography and OCT. Our method incorporated several innovative methodological approaches, rendering it to an easily applicable, versatile, reproducible, time-efficient, and user-friendly tool. Our technique can be used in the clinical setting to provide information about the bifurcation anatomy and plaque burden, thereby enabling clinical planning, education, and decision making in cardiac catheterization laboratory.

## Supplementary information

Supplementary file1
