# Supplementary material for: 3D reconstruction of coronary artery bifurcations from coronary angiography and optical coherence tomography: feasibility, validation, and reproducibility
Source: Sci Rep. 2020 Oct 22;10:18049. doi: 10.1038/s41598-020-74264-w (PMC7582159; doi:10.1038/s41598-020-74264-w)
Supplement: Supplementary file 1 — Supplementary file1 [file 41598_2020_74264_MOESM1_ESM.docx]

**Supplementary Information**

**3D Reconstruction of Coronary Artery Bifurcations from** **Coronary Angiography and Optical Coherence Tomography:** ***Feasibility, Validation, and Reproducibility***

Wei Wu, PhD^1*^; Saurabhi Samant, MBBS^1*^; Gijs de Zwart, MSc^2^; Shijia Zhao, PhD^1^; Behram Khan, MBBS^1^; Mansoor Ahmad, MBBS^1^; Marco Bologna MS^3^; Yusuke Watanabe, MD^4^; Yoshinobu Murasato, MD, PhD^5^; Francesco Burzotta, MD^6^; Emmanouil S. Brilakis, MD, PhD^7^; George Dangas, MD, PhD^8^; Yves Louvard, MD^9^; Goran Stankovic, MD^10^; Ghassan S Kassab, PhD^11^; Francesco Migliavacca, PhD^12^; Claudio Chiastra, PhD^13^; Yiannis S. Chatzizisis MD, PhD^1#^

^1^Cardiovasclar Biology and Biomechanics Laboratory, Cardiovascular Division, University of Nebraska Medical Center, Omaha, 68105, USA

^2^StudioGijs, Daendelsstraat 40, 5018 ES Tilburg, Netherland

^3^Biosignals, Bioimaging and Bioinformatics Laboratory (B3-Lab), Department of Electronics, Information and Bioengineering, Politecnico di Milano, Milan, 20133, Italy

^4^Department of Cardiology, Teikyo University Hospital, Tokyo, 173-0003, Japan

^5^Department of Cardiology, National Hospital Organization Kyushu Medical Center, Fukuoka, 810-0065, Japan

^6^Department of Cardiovascular Sciences, Fondazione Policlinico Universitario A. Gemelli IRCCS Università Cattolica del Sacro Cuore, Rome, 00168, Italy

^7^Minneapolis Heart Institute, Minneapolis, 55407, USA

^8^Department of Cardiovascular Medicine, Mount Sinai Hospital, New York City, 10029, USA

^9^Institut Cardiovasculaire Paris Sud, Massy, 91300, France

^10^Department of Cardiology, Clinical Center of Serbia, 11000, Belgrade

^11^California Medical Innovation Institute, San Diego, CA, 92121, USA.

^12^Laboratory of Biological Structure Mechanics (LaBS), Department of Chemistry, Materials and Chemical Engineering “Giulio Natta,” Politecnico di Milano, Milan, 20133, Italy

^13^PoliTo^BIO^Med Lab, Department of Mechanical and Aerospace Engineering, Politecnico di Torino, Turin, 10129, Italy

^*^The first two authors contributed equally

^#^Yiannis S. Chatzizisis MD, PhD; E-mail: [ychatzizisis@icloud.com](mailto:ychatzizisis@icloud.com)

**Supplementary Figures.**

**Supplementary Fig. S1.** Lumen area along a silicone tube imaged by OCT and µCT.

**
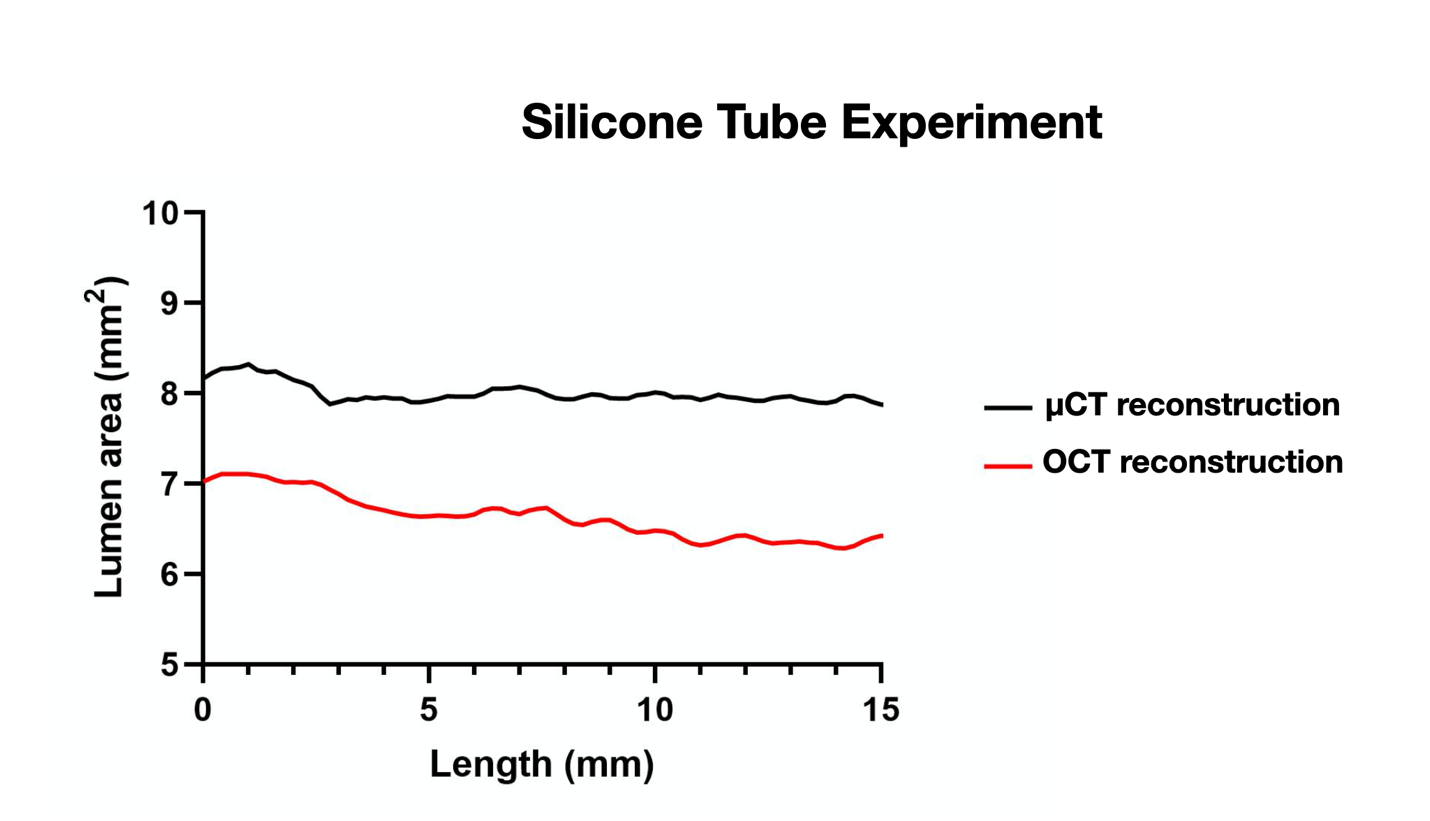
**

**Supplementary Fig. S2:** Definition of bifurcation angles: The carina points M’ and S’ were identified on the MV and SB centerlines, respectively, and connected with a straight line. The midpoint P of that straight line was connected with the merging point M of the MV and SB centerlines. The midpoint R of the straight line connecting points M and P were identified as the true carina of the bifurcation. Point R was connected with points m and s of the MV and SB centerlines, respectively, and the three bifurcation angles were calculated: Angle A between the proximal MV and SB, angle B between the distal MV and SB, and angle C between the distal and proximal MV.

**
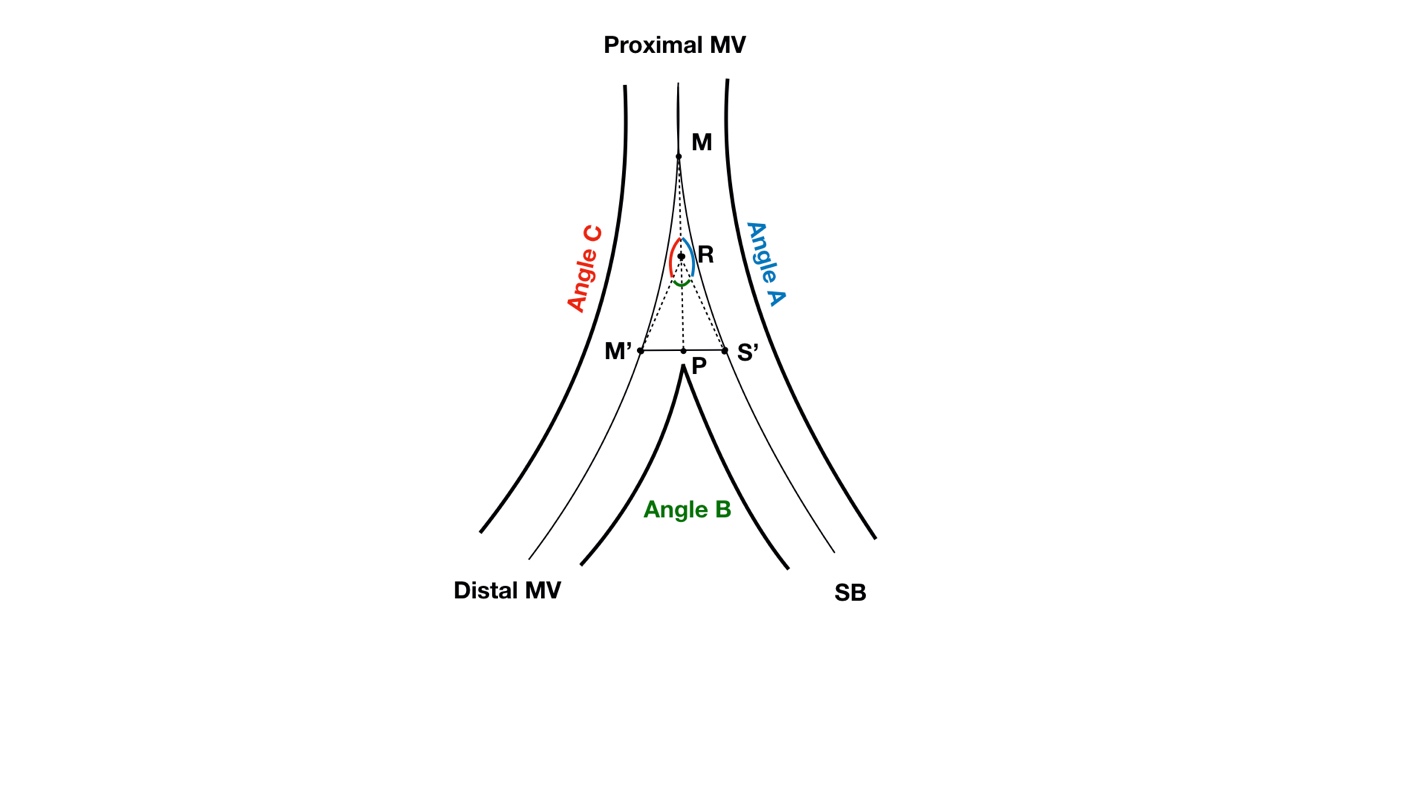
**

**Supplementary Fig. S3:** Approach for OCT wall segmentation. Two examples (a and b) showing OCT wall delineation in case of ill-defined outer wall.

**
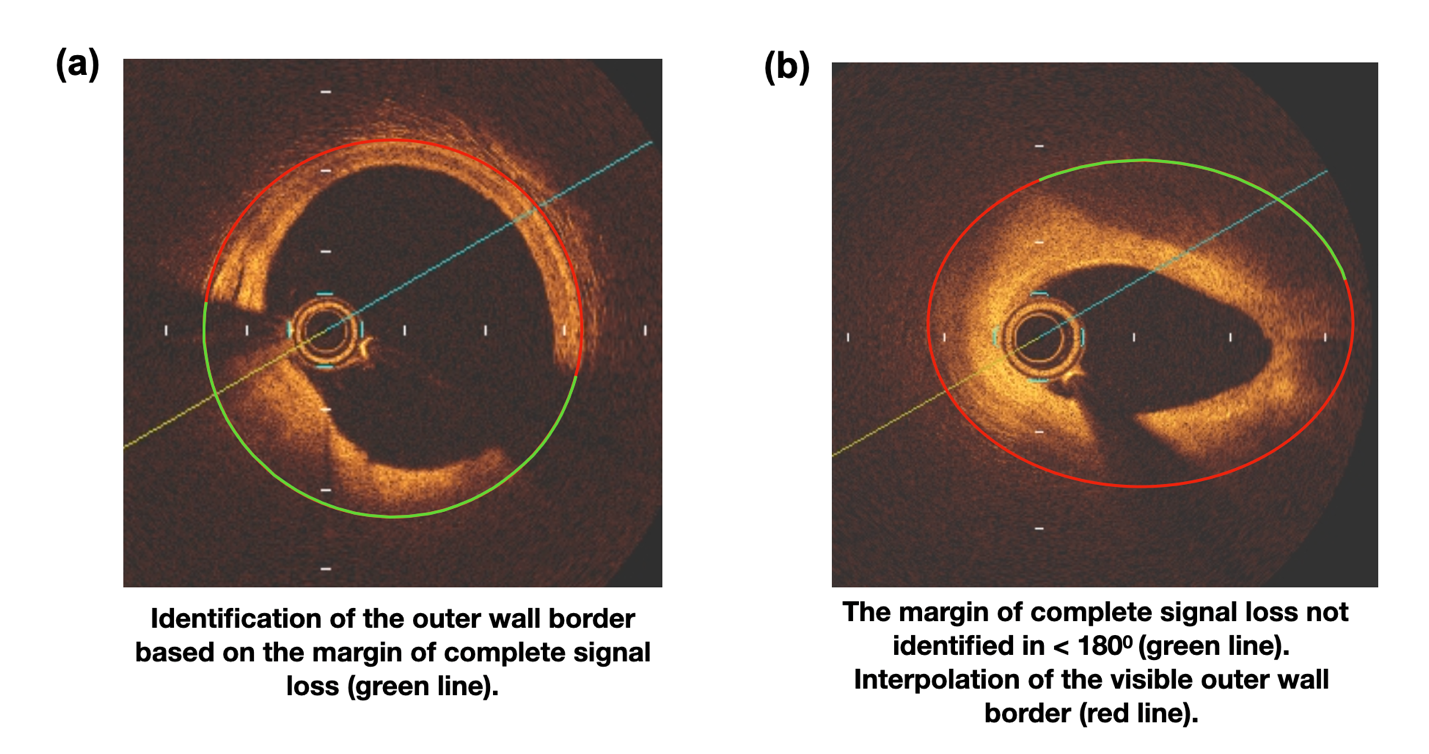
**

**Supplementary Fig. S4:** Bland-Altman analysis plots of bifurcation angles and lumen shape showing comparison between (a) 3D OCT reconstructed model vs. µCT reconstructed model (b) first and second 3D OCT reconstructed models

**
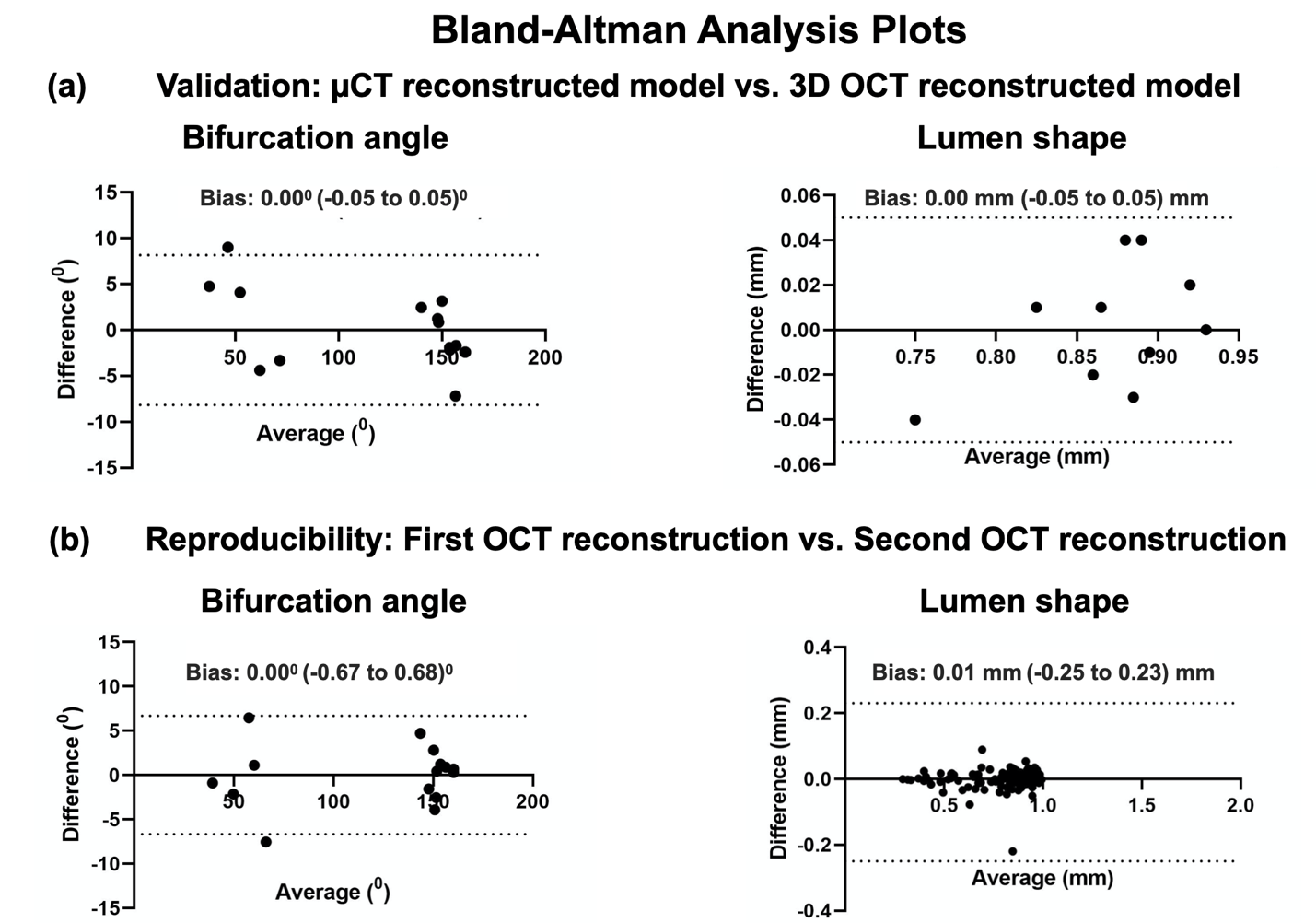
**

**Supplementary Fig. S5.** 3D reconstruction of 6 patient bifurcations (lumen and wall) using OCT and angiography.

**
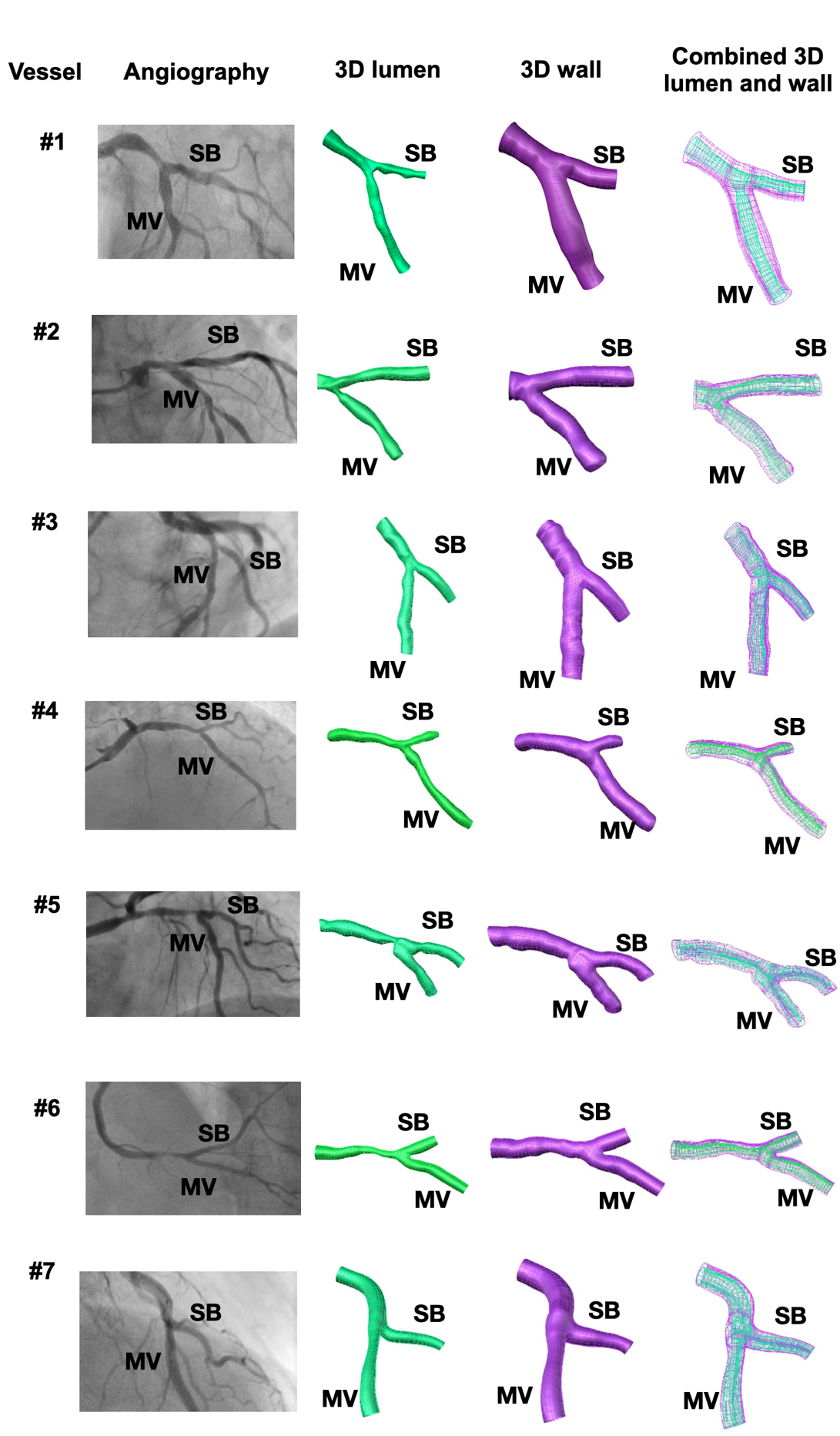
**

**Supplemental Tables.**

**Supplementary Table S1.** Patient-specific silicone and real patient coronary bifurcations used in validation, reproducibility and feasibility studies

| Vessel number | Coronary bifurcation | |
| --- | --- | --- |
| Silicone | **Main vessel** | **Side branch** |
| #1 | Left anterior descending | First diagonal |
| #2 | Left circumflex artery | Obtuse marginal |
| #3 | Left circumflex artery | Third obtuse marginal |
| #4 | Left anterior descending | First diagonal |
| #5 | Left anterior descending | Second diagonal |
| Patient | **Main vessel** | **Side branch** |
| #1 | Left anterior descending | Left circumflex artery |
| #2 | Left anterior descending | Left circumflex artery |
| #3 | Left anterior descending | First diagonal |
| #4 | Left anterior descending | First diagonal |
| #5 | Left anterior descending | First diagonal |
| #6 | Right coronary artery | Posterolateral branch |
| #7 | Left anterior descending | Second diagonal |

**Supplementary Table S2.** Bland Altman analysis showing the mean differences in bifurcation angles (A, B and C) between the 3D OCT- vs. µCT-reconstructed models.

| Bifurcation Angles | Mean difference (degrees) | 95% Limits of Agreement (degrees) |
| --- | --- | --- |
| Angle A | 1.62 | -5.77, 9.01 |
| Angle B | 2.03 | -9.15, 13.22 |
| Angle C | 0.40 | -4.22, 5.02 |

**Supplementary Table S3**. Reproducibility of lumen shape (distance Y/distance X ratio) in silicone models.

| Models | | First OCT reconstruction | | Second OCT reconstruction | |
| --- | --- | --- | --- | --- | --- |
|  |  | Median | 25^th,^ 75^th^ percentile | Median | 25^th^, 75^th^ percentile |
| #1 | MV | 0.85 | 0.66, 0.91 | 0.88 | 0.66, 0.91 |
|  | SB | 0.87 | 0.74, 0.95 | 0.87 | 0.74, 0.96 |
| #2 | MV | 0.82 | 0.72, 0.88 | 0.83 | 0.73, 0.87 |
|  | SB | 0.87 | 0.77, 0.94 | 0.86 | 0.77, 0.95 |
| #3 | MV | 0.92 | 0.86, 0.93 | 0.91 | 0.86, 0.93 |
|  | SB | 0.91 | 0.81, 0.93 | 0.91 | 0.85, 0.92 |
| #4 | MV | 0.87 | 0.73, 0.96 | 0.86 | 0.71, 0.95 |
|  | SB | 0.85 | 0.80, 0.91 | 0.84 | 0.78, 0.90 |
| #5 | MV | 0.78 | 0.62, 0.91 | 0.78 | 0.59, 0.92 |
|  | SB | 0.91 | 0.69, 0.94 | 0.90 | 0.73, 0.54 |

**Supplementary Table S4**. Reproducibility of bifurcation angles in silicone models. Bland Altman analysis showing the mean differences of bifurcation angles (A, B, C) between the two OCT reconstructed models.

| Bifurcation  Angles | Bias (degrees) | 95% Limits of Agreement  (degrees) |
| --- | --- | --- |
| Angle A | 0.43 | -5.09, 5.95 |
| Angle B | 0.59 | -10.55, 9.36 |
| Angle C | 0.17 | -4.68, 5.03 |
